# Real-Time PCR in HIV/*Trypanosoma cruzi* Coinfection with and without Chagas Disease Reactivation: Association with HIV Viral Load and CD4^+^ Level

**DOI:** 10.1371/journal.pntd.0001277

**Published:** 2011-08-30

**Authors:** Vera Lúcia Teixeira de Freitas, Sheila Cristina Vicente da Silva, Ana Marli Sartori, Rita Cristina Bezerra, Elizabeth Visone Nunes Westphalen, Tatiane Decaris Molina, Antonio R. L. Teixeira, Karim Yaqub Ibrahim, Maria Aparecida Shikanai-Yasuda

**Affiliations:** 1 Department of Infectious and Parasitic Diseases, Faculdade de Medicina, University of São Paulo (FMUSP), São Paulo, Brazil; 2 Laboratory of Immunology, Hospital das Clínicas, FMUSP, São Paulo, Brazil; 3 Division of Clinics of Infectious and Parasitic Diseases, Hospital das Clínicas, FMUSP, São Paulo, Brazil; 4 Laboratory of Parasitology, Hospital das Clínicas, FMUSP, São Paulo, Brazil; 5 Instituto Adolfo Lutz, São Paulo, Brazil; 6 Laboratório Multidisciplinar de Pesquisa em Doença de Chagas, Universidade de Brasília, Brasília, Brazil; The George Washington University Medical Center, United States of America

## Abstract

**Background:**

Reactivation of chronic Chagas disease, which occurs in approximately 20% of patients coinfected with HIV/*Trypanosoma cruzi (T. cruzi)*, is commonly characterized by severe meningoencephalitis and myocarditis. The use of quantitative molecular tests to monitor Chagas disease reactivation was analyzed.

**Methodology:**

Polymerase chain reaction (PCR) of kDNA sequences, competitive (C-) PCR and real-time quantitative (q) PCR were compared with blood cultures and xenodiagnosis in samples from 91 patients (57 patients with chronic Chagas disease and 34 with HIV/*T. cruzi* coinfection), of whom 5 had reactivation of Chagas disease and 29 did not.

**Principal Findings:**

qRT-PCR showed significant differences between groups; the highest parasitemia was observed in patients infected with HIV/*T. cruzi* with Chagas disease reactivation (median 1428.90 *T. cruzi*/mL), followed by patients with HIV/*T. cruzi* infection without reactivation (median 1.57 *T. cruzi*/mL) and patients with Chagas disease without HIV (median 0.00 *T. cruzi*/mL). Spearman's correlation coefficient showed that xenodiagnosis was correlated with blood culture, C-PCR and qRT-PCR. A stronger Spearman correlation index was found between C-PCR and qRT-PCR, the number of parasites and the HIV viral load, expressed as the number of CD4^+^ cells or the CD4^+^/CD8^+^ ratio.

**Conclusions:**

qRT-PCR distinguished the groups of HIV/*T. cruzi* coinfected patients with and without reactivation. Therefore, this new method of qRT-PCR is proposed as a tool for prospective studies to analyze the importance of parasitemia (persistent and/or increased) as a criterion for recommending pre-emptive therapy in patients with chronic Chagas disease with HIV infection or immunosuppression. As seen in this study, an increase in HIV viral load and decreases in the number of CD4^+^ cells/mm^3^ and the CD4^+^/CD8^+^ ratio were identified as cofactors for increased parasitemia that can be used to target the introduction of early, pre-emptive therapy.

## Author Summary

Chagas disease is endemic in Latin America and is caused by the flagellate protozoan *T. cruzi*. The acute phase is asymptomatic in the majority of the cases and rarely causes inflammation of the heart or the central nervous system. Most infected patients progress to a chronic phase, characterized by cardiac or digestive involvement when not asymptomatic. However, when patients are also exposed to an immunosuppressant (such as chemotherapy), neoplasia, or other infections such as HIV, *T. cruzi* infection may develop into a severe disease (Chagas disease reactivation) involving the heart and central nervous system. The current microscopic methods for diagnosing Chagas disease reactivation are not sensitive enough to prevent the high rate of death observed in these cases. Therefore, we propose a quantitative method to monitor blood levels of the parasite, which will allow therapy to be administered as early as possible, even if the patient has not yet presented symptoms.

## Introduction

Chagas disease is endemic in Latin America, where fewer than 8 million people, many of whom live in urban centers, are infected by *T. cruzi* [1]. In Brazil, the control of the Chagas disease insect vector *Triatoma infestans* and prevention of the transmission of *T. cruzi* parasitosis by blood transfusion have led to epidemiologic changes, shifting the predominant *T. cruzi* transmission routes to oral, congenital, and organ transplant transmission. HIV/*T. cruzi* coinfection has been found in urban centers, and HIV infection [2] has spread to regions in which Chagas disease is endemic. In addition, Chagas disease is now an emerging disease in developed countries, with active congenital and organ transplant transmission and reactivation of the chronic disease [3], [4].

Acute Chagas disease is characterized by high levels of parasitemia, which is detected by direct microscopy of fresh buffy coat, a quantitative buffy coat (QBC) test, or a microhematocrit test [5], [6]. In the chronic disease, low-level parasitemia is observed and can be detected only by indirect parasitological methods (xenodiagnosis and blood culture) [7]. Anti-*T. cruzi* IgG antibodies are found in almost 100% of these patients [8]. Most chronically infected patients do not develop clinical symptoms of Chagas disease, but approximately 20–30% suffer from heart and or digestive tract disease [8].

*T. cruzi* parasites are detected more frequently and with higher parasitemia levels in HIV coinfected patients than in those with chronic Chagas disease alone [9], [10]. Reactivation of chronic Chagas disease, which occurs in approximately 20% of individuals coinfected with HIV/*T. cruzi*, is characterized by high parasitemia levels, similar to an acute infection [11]. More severe disease (meningoencephalitis and/or myocarditis) has been commonly described in patients infected with HIV/*T. cruzi* [12]–[15]; the involvement of other organs, such as the skin [16], gastrointestinal tract, and pericardium, has also been reported [14]. The diagnosis of Chagas disease reactivation is based on direct observation methods [5], [6]. However, this diagnosis is not usually made during the early phase of reactivation, and many patients die soon after diagnosis or during treatment [11]. Case fatality is higher in patients with late diagnosis of reactivation because they die soon after the introduction of the therapy [6], [8]–[12].

Sensitive and rapid methods are required to monitor parasitemia in immunosuppressed patients with Chagas disease. Xenodiagnosis and blood culture are highly sensitive for the acute disease but are labor-intensive and time-consuming methods and the results take 30–120 days to be analyzed. In addition, technical expertise is required to manipulate live parasites, due to the risk of infection of laboratory staff [7], [9], [17]. In HIV/*T. cruzi*-infected patients, semi-quantitative xenodiagnosis that indicates the percentage of nymphs per assay may predict the occurrence of Chagas disease reactivation. Episodes of reactivation have been observed in 50% of patients who show ≥20% nymphs per assay in a follow-up period of 5 years [11].

A competitive polymerase chain reaction (C-PCR) method [18], [19] has been reported for monitoring the treatment of children with congenital Chagas disease, patients with chronic Chagas heart disease, and a patient with HIV and meningoencephalitis. It was used to demonstrate clearance or early detection of the parasite [19]–[21]. Another molecular method, quantitative real-time PCR (qRT-PCR), has been used to diagnose congenital [22], [23] and chronic Chagas disease and showed 41% positive detection of the chronic disease [22]. Using this method, low parasitemia was found in mothers (93.3%<10 parasites/mL) and higher parasitemia was found in neonates (76.3%>1,000 parasites/mL) [23]; parasitemia correlated negatively with age (0.01–640 parasites/mL) [24].

The aim of this study was to evaluate the use of a new quantitative molecular method (qRT-PCR) to monitor *T. cruzi* parasitemia in HIV-infected patients with or without Chagas disease reactivation. In addition, the sensitivities of different molecular and parasitological tests were compared.

## Methods

### Participants

The study included 91 samples that were collected between 1996 and 2008 from patients ≥18 years old with Chagas disease who were admitted to the AIDS Clinic and/or Clinic of Infectious and Parasitic Diseases at the Hospital das Clinicas, a tertiary hospital attached to the School of Medicine of the University of São Paulo, Brazil. The patients were classified into two groups: (1) 57 immunocompetent patients with chronic Chagas disease (CR) and (2) 34 patients with chronic Chagas disease coinfected with HIV, of whom 29 lacked reactivation (CO) and 5 had reactivation of Chagas disease (RE). The inclusion criterion for patients with Chagas disease with or without HIV infection was the presence of antibodies in two or three conventional serological tests for Chagas disease (indirect immunofluorescence (≥1/40), indirect hemagglutination (≥1/40) or Enzyme linked immunoassay (ELISA)) [24]. HIV patients were included after detection of anti-HIV antibodies by ELISA and confirmation by immunoblot [25]. Chagas disease reactivation was diagnosed if at least one of the following tests was positive: direct blood microscopy or QBC for *T. cruzi* (two patients) or direct cerebrospinal fluid (CSF) examination for *T. cruzi* (three patients). A control group of 58 healthy individuals without Chagas disease (indicated by negative conventional serological tests for Chagas disease) was used to check for contamination during the sample extraction process; the control samples were paired with samples from patients.

### Direct and indirect parasitological assays

Trypomastigotes were identified by direct microscopy of peripheral blood mononuclear cells (PBMCs) or through QBC analysis [6]. For QBC, the blood was collected in a microhematocrit tube containing acridine orange (BD Biosciences). After centrifugation, the parasites remaining in the platelet layer at the top of the buffy coat were identified by immunofluorescence microscopy. The blood culture assay was performed as previously described [17]. Six culture tubes were examined after 10, 20, 30, 60, 90 and 120 days of culture. The results are expressed as the number of positive tubes divided by the total number of tubes examined (% positive tubes); the result was considered positive if any tube was positive and negative if all were negative. Xenodiagnosis was performed with 20–40 nymphs of *T. infestans* fed in vitro with 10 mL of patient blood. The search for *T. cruzi* in the gut contents of each triatome was performed 30, 60 and 90 days later and the results are expressed as the percentage of positive insects (semi-quantitative xenodiagnosis); or a positive result if at least one insect was positive and negative if all of them were negative [7], [26].

### Sample preparation and DNA extraction

DNA was extracted with QIAamp™ DNA Mini Kit (Qiagen, Hilden, Germany) from whole blood collected in 6 M guanidine HCl plus 0.2 M EDTA buffer (pH 8); in a few cases, DNA was extracted from blood collected in EDTA (PBMC) or from CSF, which was collected from two patients with central nervous system reactivation, as previously reported [27], [28]; samples were stored at −20°C. The quantity and purity of the DNA were determined with a spectrophotometer (Gene-Quant, Pharmacia Biotech, Cambridge, England), and only samples with high purity were used in the experiments.

### Qualitative PCR

PCR was performed using the S35 and S36 primer pair, which amplifies a 330-bp minicircle sequence from *T. cruzi* (Gibco™ Life Technologies, CA, USA) [29]. The reactions contained *Taq* polymerase, 0.2 µM of each primer, 1.4 mM MgCl_2_ and 50–150 ng of DNA. Negative controls for the master mix preparation and DNA addition and a positive control, which consisted of 2×10^−15^ µg of DNA from the Y strain of *T. cruzi*, were used. The presence of inhibitors of DNA amplification was verified by β-actin amplification and by amplification of duplicate patient samples containing parasite DNA. To assess the analytical sensitivity of the qualitative PCR assays, 10-fold dilutions from 0.2 pg to 0.002 fg of DNA parasites were processed; the detection limit of the assay was 0.2 fg of *T. cruzi*, which corresponded to 0.01 parasite/assay in an agarose gel.

### Competitive PCR

A 280 bp DNA fragment with binding sites for the S34/S35 oligonucleotides was used for competitive PCR with the kDNA 330 bp product and was cloned into the pT7 Blue vector (kindly provided by the Laboratório Multidisciplinar de Pesquisa em Doença de Chagas, Universidade de Brasília). The assay was performed in the Laboratory of Immunology, Faculdade de Medicina da USP, as previously described [18]. Known concentrations of the competitor (150, 15, 1.5 and 0.15 fg), or no competitor, were mixed with four aliquots of DNA from patient samples that had previously shown positive PCR results for kDNA. For each competitive PCR analysis, we included five samples per patient.

The equivalence point was determined by visually comparing the intensities of the 280 and 330 bp products. The number of *T. cruzi*/mL of blood was calculated based on the blood volume used for extraction, the dilution of the sample, and the amount of patient DNA used in the PCR reaction.

### Real-time PCR

PCR for the microsatellite sequence TCZ3/TCZ4 (TGCTGCAGTCGGCTGATCGTTTTCGA/CAAGCTTGTTTGGTGTCCAGTGTGTGA), which was previously described by Ochs et al. (1996) [30] as internal primers for TCZ1 and TCZ2 (Gibco™ Life Technologies, CA, USA), was performed using 20 µl SYBR™ Advantage™ qRT-PCR Premix (Clontech, CA, USA), according to the manufacturer's instructions. The mixture contains *Taq* polymerase, 0.2 µM of each primer, 1.4 mM MgCl_2_ and 50–150 ng of DNA. We amplified a 149 bp sequence using 45 PCR cycles with a denaturation temperature of 94°C, an annealing temperature of 64°C and an extension temperature of 72°C on a RotorGene 3000™ (Corbett Research, Australia).

All DNA extractions and amplification reactions were performed with the appropriate negative controls to detect contamination at any stage of the procedure and with positive controls that gave reproducible results during all of the experiments.

**Figure 1:**
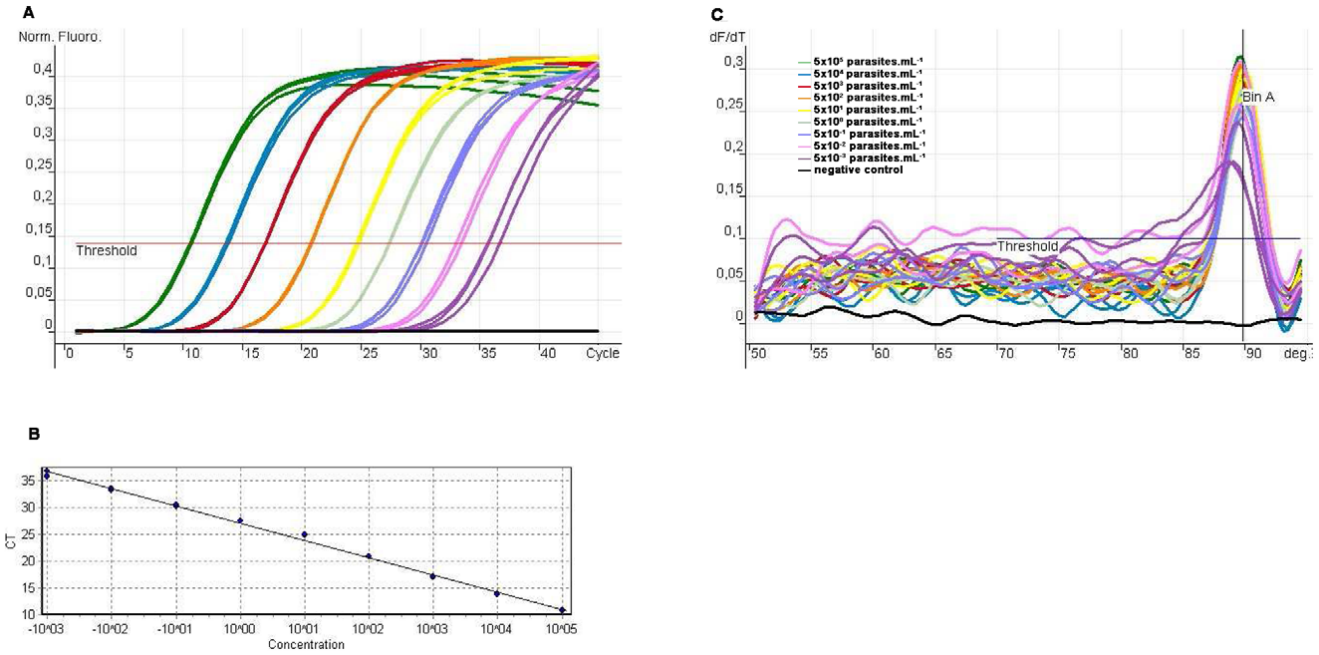
Standardization of qRT-PCR using the SYBR Green system. **A)** Standard amplification curve generated by10 fold serial dilutions of DNA from blood spiked with *T. cruzi* epimastigotes DNA (from 5.10^4^ to 5.10^−3^ parasites/mL) and negative controls, threshold = 0.12, efficiency = 1.05. **C)** Linear regression curve and regression coefficient, *R* ^2^ = 0.986. **B)**. Melting curve analysis of amplicons of samples represented in A: Tm = 89.66±0.25°C.

The standard amplification curve was prepared from 10-fold dilutions of DNA from blood spiked with 5×10^5^ to 5×10^−3^ *T. cruzi* epimastigotes/mL. Y strain (human, Brazil). The detection limit was found at 0.005 parasite/mL (Figure 1). The initial number of parasites (10^7^/mL) was counted by microscopic examination (Neubauer camera). The first blood aliquot mixed with Guanidine HCl-EDTA (v/v) was spiked with 5.10^5^ parasites cells/mL. After homogenization, this tube was used as starter for preparing 10 ten-fold serial dilutions ranging from 5×10^5^ to 5×10^−3^ *T. cruzi* epimastigotes/mL.

The final concentration of the patient sample was calculated based on the volume of the blood extracted, the amount of DNA amplified and the volume and dilution of the sample analyzed. Dilution of the samples was necessary for patients with reactivation and for some coinfected patients with large numbers of parasites.

High levels of parasitemia were used for comparison among the different methods or groups but were not necessarily indicative of reactivation. Three trained individuals performed and read the tests (one was responsible for the xenodiagnosis, one for the hemoculture and the other for the molecular tests). The results were read blind.

### Viral load

The HIV plasma viral load was determined by reverse-transcriptase (RT)-PCR using an Amplicor™ HIV-1 Monitor Test (Roche Diagnostic Systems, NJ, USA) in the Central Laboratory of Hospital das Clínicas da Faculdade de Medicina da Universidade de São Paulo, which had a lower detection limit of 200 copies of HIV/mm^3^.

### CD4^+^ and CD8^+^ counts

CD4^+^ and CD8^+^ T lymphocyte counts were determined by flow cytometry (FACSCalibur, BD Biosciences, CA, USA) using antibodies to CD4 and CD8 (BD Biosciences).

### Ethics

All protocols were approved by the ethical committee (Comissão de Ética para Análise de Projetos de Pesquisa - CAPPesq), Hospital das Clínicas, Faculdade de Medicina of University of São Paulo. Written informed consent was obtained from all participants.

### Statistical analysis

SPSS version 17 was used for the statistical analyses. The chi-squared test and Fisher exact test were used to compare the qualitative results of the tests and the differences between the patient groups. The McNemar test was used for comparisons between paired proportions. For non-parametric data, Spearman's rank correlation coefficient was used for quantitative variables (*P*<0.01) [31]. Kruskal–Wallis one-way analysis of variance was also applied to non-parametric data to compare the three groups of patients, followed by the comparison of independent groups, two by two, by the Dunn's Multiple Comparison post test (Prism 3.0, Graph Pad Software Incorporation, CA, California). *P* values<0.05 were considered significant.

## Results

### Demographic characteristics

There were no significant differences in gender (CR male frequency = 38.6%; CO male frequency = 48.3%; RE male frequency = 60.0%) among the groups with and without HIV infection. The five individuals with chronic Chagas disease, HIV infection and reactivation of Chagas disease were younger than those without HIV infection (CR mean = 46.1±12.2 years, median = 43; CO mean = 42.0±10.1, median = 38; RE mean = 32.7±3.93, median = 33) (*P* = 0.014).

### Qualitative tests: PCR, blood culture, and xenodiagnosis

A comparison of the different tests for the HIV-positive and HIV-negative patients is shown in Table 1. PCR with the S35/36 primers was more sensitive than blood culture, xenodiagnosis and both tests combined (*P*<0.001). Patients with HIV infection (CO+RE) showed higher rates of positive results by PCR (*P*<0.001, Fisher exact test), xenodiagnosis (*P*<0.001, Fisher exact test) or both tests (*P* = 0.002, χ^2^) compared with patients without HIV infection. The highest mean percentage of positive nymphs per assay ± SD (Table 2) was observed in the RE group (Kruskal-Wallis, *P* = 0.001).

**Table 1:** Sensitivities of the qualitative PCR^a^, xenodiagnosis^b^ and blood culture^c^ in Chagas disease with/without HIV infection.

Group^d^	S35/36 PCR, % (*n*)	XD, % (*n*)	BC, % (*n*)	(XD and/or BC), % (*n*)
CR	50.9 (57)	9.3 (54)	31.6 (57)	33.3 (57)
CO	89.7 (29)	44.8 (29)	35.7 (28)	58.6 (29)
RE	100.0 (5)	60.0 (5)	100.0 (5)	100.0 (5)
Total	65.9 (91)	23.9 (88)	36.7 (90)	54.9 (91)

a qualitative S35/36 PCR,

b xenodiagnosis (XD),

c blood culture (BC),

d groups of patients: chronic Chagas disease with (CO) and without HIV infection (CR) and reactivation of Chagas disease in HIV infected patients (RE). Xenodiagnosis was not performed on three samples from chronic chagasic patients. Blood culture was not performed on one sample from co-infected patient.

**Table 2:** Results of xenodiagnosis^a^, C-PCR^b^ and qRT-PCR^c^ in the three groups of patients^d^ (CO, CR, RE).

Variable	CR	CO	RE
aXD mean % of nymphs ± SD	1.15±4.02	3.54±6.10	31.70±36.53
XD median % of nymphs	0.0	0.0	24.3
XD min-max % nymphs	0.0–21.0	0.0–28.6	0.0–86.7
C-PCR mean n^o^ of parasites ± SD	20.73±30.74	964.06±1685.82	16500.00±19006.58
C-PCR median n^o^ of parasites	14.07	175.00	10000.00
C-PCR min-max n^o^ of parasites	0.0–125.0	0.0–5000.0	5000.0–50000.0
qRT-PCR CT mean ± SD	33.83±0.49	31.41±0.70	24.30±2.12
qRT-PCR CT min-max	28.29–37.30	25.30–37.10	18.36–31.38
qRT-PCR mean n^o^ of parasites ± SD	0.44±0.20	10.43±3.53	12584.96±11368.35
qRT-PCR median n^o^ of parasites	0.00	1.57	1428.90
qRT-PCR min-max n^o^ of parasites	0.00–10.98	0.00–70.00	109.20–58000.00

a XD - Xenodiagnosis (% of nymphs positive per assay) was performed in 90 patients,

b C-PCR (number of parasites) in 38 patients and

c qRT-PCR in 91 patients (Ct for 53 positive samples and number of parasites for 91 patients = 91 samples),

d groups of patients: chronic Chagas disease with (CO, 29 patients) and without HIV infection (CR, 57 patients) and reactivation of Chagas disease in HIV infected patients (RE, 5 patients).

### Quantitative tests

#### Competitive PCR

**Figure 2:**
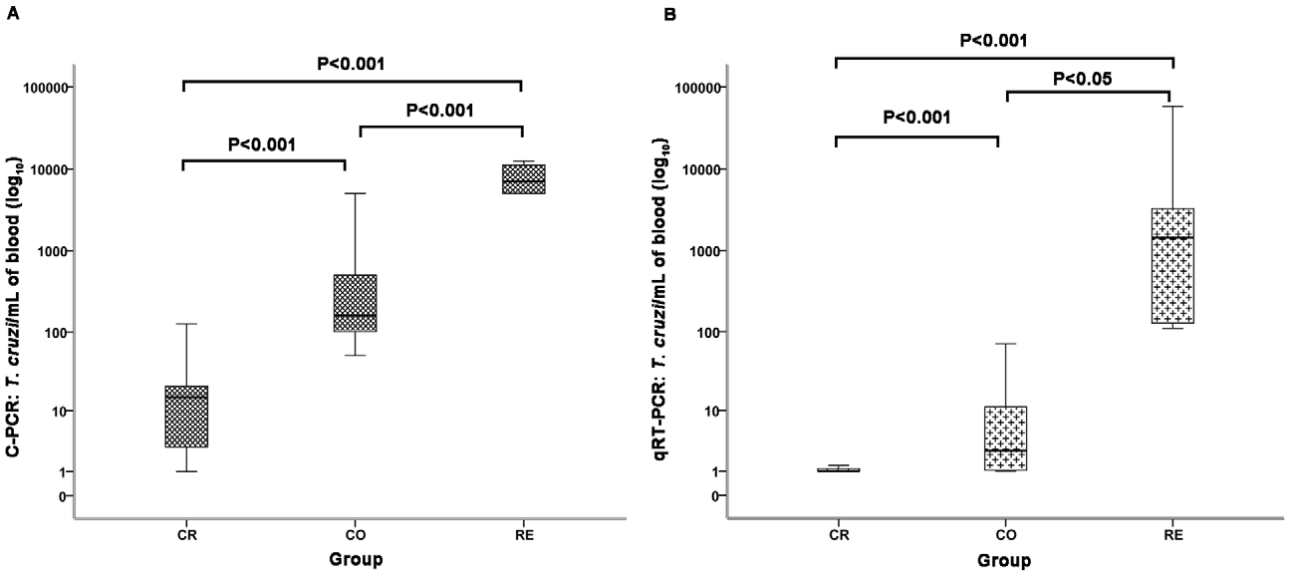
C-PCR and qRT-PCR in the blood of patients' groups (CR, CO and RE). **A)** Number of *T. cruzi*/mL (log_10_) in blood by C-PCR. CR (*n* = 17); CO (*n* = 16) and RE (*n* = 5). The line represents the median. Comparative analysis among the groups showed a statistically significant difference (*P*<0.001) by Kruskal–Wallis test. Comparing the groups, a statistically significant difference was observed between CR×CO, *P*<0.001; CR×RE, *P*<0.001; and CO×RE, *P*>0.05) (Dunn s Multiple Comparison test). **B)** Number of *T. cruzi*/mL (log_10_) in blood by qRT-PCR. CR (*n* = 57); CO (*n* = 29) and RE (*n* = 5). Comparison of the qRT-PCR results observed in the three groups of patients with Chagas disease. The results revealed a statistically significant difference (*P*<0.001) by Kruskal–Wallis test, and comparison between the groups showed statistically significant differences between CR×CO (*P*<0.001), CR×RE (*P*<0.001), and CO×RE, (*P*<0.05) (Dunn's Multiple Comparison test).

The semi-quantitative assay was performed on 38 samples with positive S35/36 PCR results: 17 CR, 16 CO and 5 RE (Figure 2A). A higher number of copies was observed in patients with coinfection and Chagas disease reactivation, followed by the group with both infections but no reactivation and chronic Chagas disease patients without HIV infection (Table 2). The test was not performed on all samples because it requires a large amount of purified DNA, which was not always available in the amount necessary for the second measurement required when the equivalence point could not be determined.

#### Real-time PCR

The standard amplification curve was prepared from 10-fold dilutions of DNA from blood spiked with 5×10^5^ to 5×10^−3^ epimastigotes/mL. Figure 1 A shows the limit of detection 0.005 parasite/mL, found by Probit analysis; the slope was −3.2, the reaction efficiency was 1.05, and the linear regression curve showed *R2* = 0.986 (Figure 1B) and *T*_m_ = 89.66±0.25°C (Figure 1C). This assay was reproducible at ≥0.005 parasite/mL, as observed by Ct and Tm values in 5 different assays using triplicates and duplicates. Amplification products from patient samples with low parasitemia and doubtful melting temperature were analyzed by agarose gel electrophoresis to check the size of the expected 149 bp product (data not shown).

All 91 patients with Chagas disease with or without HIV infection were analyzed by qualitative PCR and qRT-PCR (one sample per patient) – Table 1, 2 and 3. Forty nine patients showed positive results by qualitative and quantitative PCR. A comparison between the qualitative PCR and qRT-PCR results showed undetectable parasitemia in 12.09% of samples by qRT-PCR and 4.39% only by S35/S36 PCR despite the positive result by other test.

**Table 3:** Spearman's rank correlation coefficients observed between tests: blood culture, xenodiagnosis, competitive PCR, quantitative real-time PCR.

Methods (quantification method)^a^	Spearman *r*_s_	*P*	Samples (*n*)
XD×BC^b^ (% nymphs +)×(% tubes +)	0.398	<0.001	87
C-PCR×XD^b^ (*T. cruzi*/mL)×(% nymphs +)	0.456	0.006	35
C-PCR×BC (*T. cruzi*/mL)×(% tubes +)	0.270	0.106	37
qRT-PCR^c^×XD (*T. cruzi*/mL)×(% nymphs +)	0.457	<0.001	88
qRT-PCR^c^×BC (*T. cruzi*/mL)×(% tubes +)	0.473	<0.001	90
qRT-PCR^c^×C-PCR^b^ (*T. cruzi*/mL)×(*T. cruzi*/mL)	0.725	<0.001	38
qRT-PCR^c^×viral load^b^ (*T. cruzi*/mL)×(RNA copies/mL)	0.584	0.007	20
qRT-PCR^c^×CD4^+^ T cells^b^ (*T. cruzi*/mL)×(cells/mm^3^)	−0.381	0.038	30
qRT-PCR^c^×CD4^+^/CD8^+^ b (*T. cruzi*/mL)×(cells/mm^3^)	−0.484	0.007	30

a Blood culture (BC), xenodiagnosis (XD), competitive PCR (C-PCR), and quantitative real-time PCR (qRT-PCR).

b Correlation is significant at the 0.01 level (2-tailed).

c qRT-PCR was performed only on positive S35–S36 PCR samples.

The level of parasitemia, expressed as DNA copies/mL, was highest in the RE group, followed by CO and CR (Figure 2B, Table 2). In the CSF of two patients with HIV/*T. cruzi* coinfection and Chagas disease reactivation, the number of parasite copies was greater than 5×10^5^ copies/mL.

Patients in the HIV/*T. cruzi*–coinfected group without Chagas disease reactivation presented different levels of parasitemia (Table 2); the majority were similar to the chronic cases, but a small portion (<10.0%) had the highest levels in this group, and the remainder had intermediate levels (data not shown).

**Figure 3:**
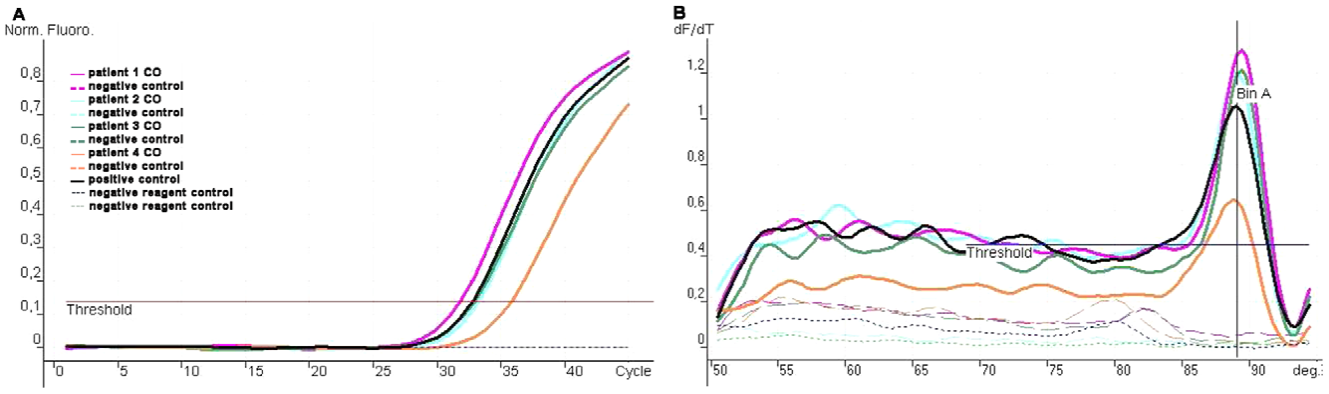
Amplification of T. cruzi DNA extracted from blood of four patients with HIV/T. cruzi coinfection (CO). Colored continuous lines represent CO patients (4.83–142.4 parasites/mL), and black line represents the positive control, 5×10^−14^ parasites/mL. **A)** Dashed lines represent the controls without Chagas disease, and the negative controls for the reagents and room of DNA application. **B)** Dashed lines represent the negative control individual without Chagas disease for each patient. Dotted lines represent the negative controls for the reagents and DNA application.

The amplification of *T. cruzi* DNA extracted from the blood of four patients with HIV/*T. cruzi* coinfection (CO) is shown in Figure 3.

### Correlation analysis of the quantitative results

**Figure 4:**
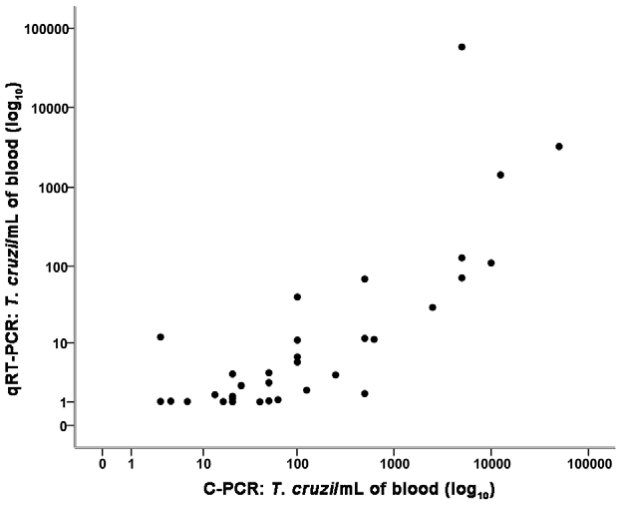
Correlation between number of parasites/mL of blood by competitive PCR (C-PCR) and real-time PCR (qRT-PCR). Correlation between number of *T. cruzi*/mL in 38 paired samples from patients infected with HIV/*T. cruzi* with or without Chagas disease reactivation. Spearman's correlation index (*r*_s_) = −0.725, *P*<0.001.

Table 3 shows the Spearman correlation indices (*r_s_*) of the quantitative molecular, parasitological and immunological tests. A strong positive correlation between the number of parasites/mL detected by C-PCR and qRT-PCR was shown in Figure 4.

**Figure 5:**
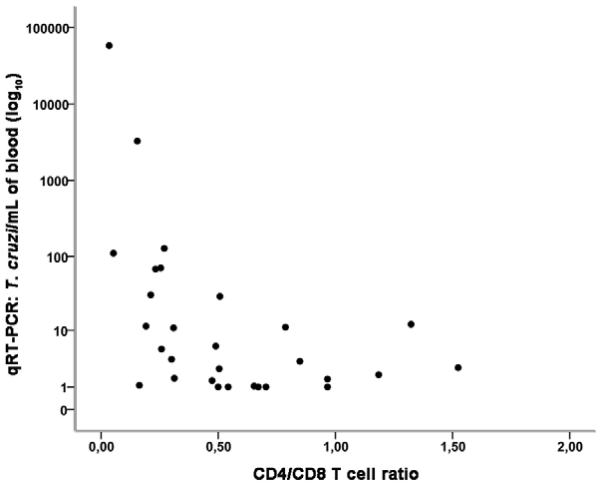
Correlation between the number of parasites/mL of blood (qRT-PCR) and CD4^+^/CD8^+^ T cell ratio. Spearman correlation index (*r*_s_) = −0.484, *P* = 0.007 by analysis of 30 samples from patients infected with HIV/*T. cruzi* with and without Chagas disease reactivation.

To study the influence of CD4^+^ and CD8^+^ T cells on the level of parasitemia, we calculated the Spearman correlation coefficient for 30 samples from patients infected with HIV/*T. cruzi* with and without parasite reactivation and found negative correlations with the number of CD4^+^ T cells (data not shown) and the CD4^+^/CD8^+^ ratio (Figure 5). However, no correlation was observed with the number of CD8^+^ T cells (data not shown).

**Figure 6:**
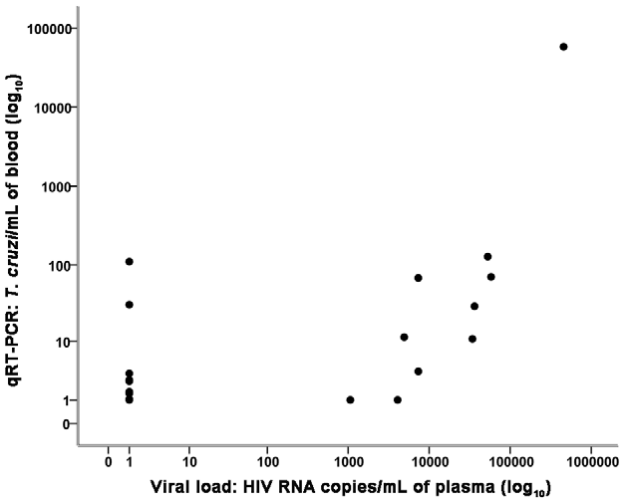
Correlation between number of T. cruzi/mL of blood (qRT-PCR) and HIV RNA copies/mL of plasma). Spearman's correlation coefficient (*r_s_*) = 0.584, *P* = 0.007 (Analysis of 20 samples from patients infected with HIV/*T. cruzi* with and without Chagas disease reactivation). In this Figure, 8 samples were superimposed in the lower limit of viral load and number of parasites/mL.

A positive correlation was found between the HIV viral load and the level of *T. cruzi* parasitemia in 20 samples from individuals infected with HIV/*T. cruzi* with and without Chagas disease reactivation (Figure 6).

## Discussion

In this study, the utility of the molecular methods C-PCR and qRT-PCR for the diagnosis and quantification of the number of parasite DNA copies in the blood of patients with HIV/*T. cruzi* coinfection was demonstrated for the first time.

In addition, we have shown that qRT-PCR was able to distinguish between the groups of HIV/*T. cruzi*–infected patients with and without Chagas disease reactivation and between groups of patients with chronic Chagas disease and those coinfected with HIV/*T. cruzi*, with or without Chagas disease reactivation (Figure 2 B and Table 2).

High levels of parasitemia have been reported in three chronic heart disease patients after heart transplantation [24], but no report has compared parasitemia in reactivated and non-reactivated HIV/*T. cruzi*-infected groups. The inclusion of the latter group indicated that there are different levels of parasitemia in these patients. The majority had similar levels to the chronic cases, less than 10% had the highest levels of the group and the remainder showed intermediate levels of parasitemia. The patients with higher parasitemia might be targeted for therapy.

Additionally, unlike what we would expect based on the immunopathogenesis of HIV/*T. cruzi* coinfection, we found that one HIV-infected patient with reactivation had much lower parasitemia than the majority of the RE group. This episode of reactivation was characterized by the presence of trypomastigotes, as detected by direct microscopy of the blood [32]. The patient only presented mild symptoms without meningoencephalitis, myocarditis or other tissular lesions. The level of CD4^+^ T cells was more than 300/mm^3^, and, during this period, the patient showed >20% of nymphs on xenodiagnosis and an increased viral load. The lineage type of the parasite was no different from the majority of the cases. It is possible that the level of parasitemia and the level of CD4^+^/mm^3^ did not change because the reactivation was diagnosed in the initial phase of the disease and early therapy with benznidazole was administered. Although the number of patients with reactivation was small, the high number of DNA copies observed in the blood (Figure 2B) or cerebrospinal fluid of the remaining RE patients with myocarditis or meningoencephalitis is impressive. In our study, qRT-PCR showed high performance with a low detection limit (0.005 parasite/mL) and good efficiency, as previously described [32]–[34]. We suggest that increasing parasitemia in subsequent examinations and/or stabilization at levels higher than previously seen in the same patient should be carefully monitored for CD4^+^ counts and viral load.

The two quantitative molecular methods, C-PCR and qRT-PCR, used in the present study were strongly correlated by Spearman's correlation coefficient (0.725), showing that both could be used for diagnostic purposes. However, C-PCR is more labor-intensive and time-consuming and requires more DNA, post-PCR processing of the amplified products and subjective analysis of the results.

The previous result of 41% positivity for chronic Chagas disease by qRT-PCR [22] is similar to the results observed in our study. In addition, qRT-PCR with the S35/S36 primers was used to measure parasitemia in neonates with congenital Chagas disease [23]; this analysis yielded similar data to those observed here in the RE group compared with the CR group using satellite sequences. Those authors reported a higher level of parasitemia (>1000 copies/mL blood) in neonates compared with their mothers with chronic Chagas disease (<10 copies/mL blood) [23]. In our study, the level of parasitemia in the RE group (median 1428.90 parasites) was higher than in the CO group (median 1.57 parasites) and CR group (median 0.00 parasite). A previous study on the reactivation of Chagas disease in heart transplant patients with positive Strout tests [24] reported a lower concentration of parasites in the blood than that shown in our study (9.07 and 468.0 parasites/mL).

Our data show that C-PCR and qRT-PCR had higher sensitivities than the parasitological tests (xenodiagnosis and blood culture) and confirmed the previously described higher sensitivity of S35/S36 PCR [35]–[37]. Moreover, PCR takes less time (a few hours) and has a low risk of infection, in contrast to the labor-intensive and time-consuming parasitological tests (30–120 days), which have high specificity but require the manipulation of live parasites. The risk of DNA contamination in molecular tests needs to be minimized by using negative controls at each stage of the analysis. The risk of contamination is lower with qRT-PCR because it employs extraction kits and excludes post-processing PCR, although the high cost is a disadvantage.

Analyses of the demographical characteristics of the different clinical groups showed no differences in terms of gender, but RE patients were younger than the other groups, possibly due to the epidemiological characteristics of HIV-infected patients in Brazil [2]. A limitation of our study was that the small number of patients with Chagas disease reactivation did not allow for age-matched controls.

An analysis of the level of parasitemia in different groups represents a good strategy to monitor the host protozoan/virus imbalance. Our data were not influenced by the lineage of the parasite, which was similar for most of the isolates (data not shown), as previously observed [24]. In our study, the parasite level was lower in the CR group and higher in the CO and RE groups, possibly due to ability of the parasite to evade the host immune response in patients without HIV infection. Cellular immunity and macrophage deficiencies in HIV infection could explain the increased parasitemia observed in HIV/*T. cruzi*-infected patients. The highest level of parasitemia was seen in the RE group, which was associated with increased HIV viral load, a decreased number of CD4^+^ cells, and a decreased CD4^+^/CD8^+^ cell ratio; these results confirm the failure of immune mechanisms in the RE group. These data are corroborated by clinical studies that showed a relationship between the reactivation of trypanosomiasis, increased HIV viral load, and decreased CD4^+^ counts in peripheral blood [11].

The correlation between HIV viral load and the concentration of parasites was demonstrated for the first time in our study, although it has been previously suggested by the relationship observed between an increased viral load and an increased rate of positive xenodiagnosis in HIV/*T. cruzi*-infected patients [11]. These data are also consistent with the rapid evolution of murine leukemia virus in mice infected by *T. cruzi* [38]. Although no relationship was found between CD8^+^ cells and the concentration of parasites, their ability to control infection via IFN-γ secretion [39] has been demonstrated previously. The strong correlation between the number of parasites and CD4^+^/CD8^+^ cells suggests that both cells play a role in the control of parasitemia. The data from a previous report [40] in mice, which showed high parasitemia in CD4^−^/CD8^+^ animals and low parasitemia and high survival in CD4+/CD8+ mice, help to explain the results of this study.

Reactivation of chronic Chagas disease or increased parasitemia has been reported in *T. cruzi*-infected patients with hematological malignancies or autoimmune diseases receiving cytotoxic or anti-inflammatory therapy with corticosteroids [4], [24], [41]. Severe reactivation of trypanosomiasis has been described in about 20% [11] of patients infected with HIV (although these data are possibly overestimated by the inclusion of one referral center). In these cases, if treatment is delayed for at least 30 days, the mortality rate of such patients is 80%, but mortality decreases to 20% for patients treated within 30 days, indicating that earlier diagnosis and treatment increase patient survival [8]–[16].

One of the limitations of this study is the cross-sectional design, which does not allow for an investigation of the evolution of parasitemia. Another limitation is the low number of HIV-infected patients with Chagas disease reactivation, which constitutes an important challenge for prospective studies. Nevertheless, we observed that the high levels of parasitemia seen in the majority of HIV-infected patients with reactivation were not found in coinfected patients without reactivation.

Considering the imbalance of host-parasite interactions in HIV/*T. cruzi*-coinfected patients and the fact that HIV infection might favor parasite growth by itself, therapy might be considered in these coinfected patients on the basis of high parasitemia and low CD4^+^ count and decreased CD4^+^/CD8^+^ ratio, even though symptoms were absent. The adverse effects of the drugs, previous immunosuppression and the immunosuppressive effects of Chagas disease, poor surveillance against neoplasia and the therapy efficacy, which is lower in the presence of low levels of parasitemia, must be taken into consideration when recommending universal therapy for any coinfected patient.

We propose that prospective multicenter studies are warranted to address important questions regarding the management of HIV/*T. cruzi* coinfection, including determining why Chagas disease reactivation occurs in some individuals with lower levels of parasitemia, characterizing the influence of parasite lineage and immune responses on reactivation, and evaluating the outcome of initiating therapy on the basis of serology [42] versus treating with pre-emptive therapy (parasitemia versus uniquely or persistently high levels of parasitemia).

Finally, in the present study, we demonstrated for the first time that qRT-PCR shows different levels of parasitemia in groups of HIV/*T. cruzi*-infected patients with and without Chagas disease reactivation. The highest concentrations of parasites were found in the latter, followed by coinfected patients and, finally, patients with chronic Chagas disease. We propose that this new test be evaluated under standardized conditions in prospective controlled studies to determine the importance of parasitemia (persistent and/or increased) as a criterion for initiating pre-emptive therapy in chronic Chagas disease patients with HIV infection or immunosuppression. The association of increased parasitemia with increased viral HIV load and a decreased CD4^+^ count and CD4^+^/CD8^+^ ratio in peripheral blood suggests that these could be analyzed as cofactors of increased parasitemia to further support any intervention.

In addition, this association (increased parasitemia, increased HIV viral load and decreased number of CD4^+^ cells/mm^3^ and decreased CD4^+^/CD8^+^ ratio) reinforces the need to monitor parasitemia using quantitative methods to determine when to start therapy for the better management of Chagas disease in patients with immunosuppression.
